# Life-stage differences in nutrient digestibility and physiological parameters in Sapsaree dogs

**DOI:** 10.3389/fvets.2026.1834119

**Published:** 2026-05-28

**Authors:** Hyunjin Kyoung, Kimoon Kim, Yonggu Kang, Jin Ho Cho, Kyeong Il Park, Jinmu Ahn, Hansol Hwang, Younghoon Kim, Min Kyu Kim, Min Young Lee, Hyeun Bum Kim, Minho Song

**Affiliations:** 1 Department of Animal Science and Biotechnology, Chungnam National University, Daejeon, Republic of Korea; 2 Department of Animal Science, Chungbuk National University, Cheongju, Republic of Korea; 3 Department of Agricultural Biotechnology, Research Institute of Agriculture and Life Science, Seoul National University, Seoul, Republic of Korea; 4 Animal Welfare Division, National Institute of Animal Science, Rural Development Administration, Wanju-gun, Republic of Korea; 5 Department of Animal Biotechnology, Dankook University, Cheonan-si, Republic of Korea

**Keywords:** biochemistry, digestibility, hematology, life stage, Sapsaree dogs

## Abstract

This study evaluated the life-stage differences in growth and fecal parameters, nutrient digestibility, serum biochemistry, and hematology of Korean Sapsaree dogs. Eighteen healthy Saparees were allotted into three groups according to age (puppy, under 1 yr old; adult, 1–7 yrs old; senior, over 7 yrs old). The study included a 7-day adaptation period and a 10-day experimental period. Body weight change was highest (*p* < 0.05) in puppies (3.87%), followed by adults and seniors (0.75% and −3.27%). Puppies and adults had greater (*p* < 0.05) daily food intake than seniors. Adults had greater (*p* < 0.05) daily fecal output than puppies and seniors. Puppies and adults had greater (*p* < 0.05) apparent total tract digestibility (ATTD) of dry matter than seniors. The ATTD of crude protein was highest (*p* < 0.05) in puppies, followed by adults and seniors (88.86%, 84.70%, and 83.12%, respectively). The ATTD of methionine was highest (*p* < 0.05) in adults, followed by seniors and puppies. Puppies had greater (*p* < 0.05) the levels of serum total protein, blood urea nitrogen, and creatinine than adults and seniors. Serum albumin level was highest (*p* < 0.05) in puppies, followed by adults and seniors (4.38, 2.45, and 1.88 g/dL, respectively). At the final day of the study, puppies had greater (*p* < 0.05) the number of white blood cells than adults and seniors. Adults had greater (*p* < 0.05) the number of red blood cells and hemoglobin level than puppies and seniors. Our findings suggest that life stage should be considered a key determinant in food formulation and nutritional management, as it influences nutrient digestibility and metabolic status.

## Introduction

The nutritional physiology of companion dogs dynamically changes throughout their life stages, reflecting differences in growth, maintenance, and aging processes. Previous studies have reported age-related differences in nutrient digestibility, gut microbiota, and physiological parameters, with evidence indicating that nutrient digestibility and energy requirements change with age, while alterations also occur in immune functions and metabolic responses (1, 2). During growth, nutritional management is necessary for tissue development, skeletal formation, and immune system maturation (2–4), while during adulthood, body tissue maintenance and stable metabolic functions are crucial. On the other hand, physiological changes occur in senior age, such as decreased digestive efficiency and changes in the gut microbial composition and immune functions (2, 5, 6). Therefore, appropriate nutritional management is essential for maintaining health and wellbeing in dogs.

Currently, recommendations for canine nutritional requirements and feed composition are provided by several organizations, including the National Research Council (NRC), the Association of American Feed Control Officials (AAFCO), and the Federation of the European Companion Animal Feed Industry (7). However, these recommendation standards may not sufficiently reflect the physiological differences among different breeds, and most studies tend to focus on limited breeds or specific life stages. Previous studies shown that nutrient digestibility decreases with age, while systemic health parameters vary across life stages (8–10). Nevertheless, direct comparisons of life-stage-related digestive physiology and metabolic characteristics may differ depending on the breed of dogs, and research is relatively lacking, particularly for native or specific breeds.

The Sapsaree is a native Korean dog breed, designated as a natural monument and recognized for its historical and cultural significance (11). Compared with commonly studied companion dog breeds, the Sapsaree exhibits distinct genetic and phenotype characteristics, including body shape, hair structure, and behavioral traits. These breed-specific characteristics may influence physiological processes such as responses to food intake and nutrient metabolism. However, information on life-stage-related physiological responses in Sapsaree dogs remains limited, especially under the same dietary conditions. This lack of information limits the development of breed-specific nutritional strategies and evidence-based health management for the Sapsaree breed.

In this study, we hypothesized that nutrient digestibility and physiological parameters differ across the life stage of Sapsaree dogs under the same dietary conditions. Therefore, the objective of this study was to evaluate growth and fecal parameters, nutrient digestibility, serum biochemistry, and hematology across different life stages in Sapsaree dogs.

## Material and methods

### Experimental animals, design, and diets

The protocol for this study was reviewed and approved by the Institutional Animal Care and Use Committee of Chungnam National University, Daejeon, South Korea (approval #: 202310A-CNU-179). Eighteen healthy Sapsaree dogs were classified into three age groups (six dogs per group): puppies (under 1 yr old; average age of 26.4 ± 2.8 wks; average body weight [BW] = 19.43 ± 2.96 kg), adults (1–7 yrs old; average age of 3.8 ± 0.8 yrs; average BW = 28.63 ± 3.48 kg), and senior (over 7 yrs old; average age of 9.8 ± 1.0 yrs; average BW = 23.83 ± 2.99 kg). Among the dogs used in this study, only one individual in the adult group was neutered, while all others were intact. All dogs were housed at the Korean Sapsaree Foundation (Gyeongsan-si, South Korea). The study lasted for a total of 17 days, with a 7-day diet adaptation period (from days −7 to 0) and 10-day experimental collection period (from days 0 to 10). A basal diet based on hydrolyzed chicken powder, brown rice, and soy protein was formulated to meet or exceed the minimum nutrient requirements recommended by the AAFCO for both growth and reproduction and adult maintenance (Table 1). In particular, crude protein and indispensable amino acids were formulated to meet growth requirements, whereas crude fat and mineral contents (calcium, phosphorous, and their ratio) were formulated to meet adult maintenance requirements, thereby ensuring adequate nutrient supply across different life stages. All dogs fed the same basal diet throughout the study. The amount of extruded food provided was calculated based on the maintenance energy requirement (MER) of each dog (puppy = 132 × BW^0.75^ × 1.5; adult = 132 × BW^0.75^; senior = 105 × BW^0.75^) based on standard equation according to the NRC (12) and was offered twice daily (09:00 and 17:00). The feeding amount was determined based on MER at the beginning of the adaptation period and kept constant throughout the study, with no adjustments according to changes in BW or body condition score (BCS). On day 0, 5 g per kg of chromium oxide (Daejung Chemicals & Metals Co., Ltd., Siheung-si, South Korea) was added to diets as an indigestible marker for nutrient digestibility (13).

**Table 1:** Composition of experimental diet for sapsaree dogs (as-fed basis).

Item	Basal det
Ingredient, %	
Hydrolyzed chicken breast	35.00
Brown rice powder	32.60
Soy protein	15.00
Tapioca starch	5.00
Canola oil	3.00
Sweet pumpkin powder	2.00
Cabbage	2.00
Carrot	1.00
Monocalcium phosphate	1.80
Calcium carbonate	1.60
Vitamin-mineral premix^a^	0.55
Tocopherol	0.05
Salt	0.40
Total	100.00
Analyzed composition	
Dry matter, %	90.15
Crude protein, %	38.82
Crude fat, %	6.65
Crude fiber, %	0.27
Crude ash, %	6.55
Nitrogen-free extract^b^, %	47.72
Calcium, %	0.78
Phosphorous, %	0.65
Gross energy, kcal/g	4.87
Metabolizable energy^c^, kcal/g	3.59

^a^Vitamin and mineral premix supplied per kg of diets: 3,500 IU vitamin A; 250 IU vitamin D3; 25 mg vitamin E; 0.052 mg vitamin K; 2.8 mg vitamin B1(thiamine); 2.6 mg vitamin B2 (riboflavin); 2 mg vitamin B6 (pyridoxine); 0.014 mg vitamin B12; 6 mg Cal-d-pantothenate; 30 mg niacin; 0.4 mg folic acid; 0.036 mg biotin; 1,000 mg taurine; 44 mg FeSO_4_; 3.8 mg MnSO_4_; 50 mg ZnSO_4_; 7.5 mg CuSO_4_; 0.18 mg Na_2_SeO_3_; 0.9 mg Ca(IO_3_)_2_.

^b^Nitrogen-free extract, % = 100 − (crude protein + crude fat + crude fiber + crude ash), calculated according to AAFCO procedures.

^c^Metabolizable energy, kcal/g = (crude protein × 3.5 kcal/g) + (crude fat × 8.5 kgcal/g) + (nitrogen-free extract × 3.5 kcal/g), calculated according to AAFCO procedures.

### Data and sample collection

Individual BW was weighed and BCS was assessed on day 0 and 10 of the experimental period using a standard method based on visual observation. The amounts of food offered and left were weighed and recorded daily. Body weight change (BWC) and daily food intake (DFI) were calculated for growth parameters. Nutrient intake on metabolic BW (MBW) basis was calculated by multiplying the individual daily dry matter intake by the analyzed nutrient concentrations on a dry matter basis, and presented relative to MBW. The BCS of each dog was evaluated using a standardized 9-point system by trained personnel (14). The BCS score ranged from 1 to 9 and are classified as underweight (BCS 1–3), ideal (BCS 4–5), or overweight (BCS 6–9) based on standard criteria (15). Fecal scores for each dog were daily monitored by visual observation during the experimental period (from day 0 to 10) using a 5-point scale (dry and crumbly feces to watery diarrhea) according to the Waltham fecal scoring system (16). The frequency of hard dry feces or diarrhea was calculated by counting cage days with a cage fecal score < 2.0 or ≥ 3.5, respectively (17). Fecal samples were collected from each dog for 4 days from day 3 to 6 during the experimental period and stored at −80°C with diet until chemical analyses. Blood samples were collected from each dog via cephalic vein using 5 mL tubes (BD Vacutainer Systems, Franklin Lakes, NJ, USA) with or without ethylenediaminetetraacetic acid (EDTA) on day 0 and 10 after a 12 h fasting period. Serum samples were obtained from non-EDTA tubes by using centrifugation at 3,000 rpm for 15 min at 4 °C and stored at −80 °C for biochemical analysis.

### Chemical analyses

The stored diet and fecal samples were dried in a forced-air oven at 70 °C 72 h and finely ground on a 1 mm screen for chemical analyses. The contents of dry matter (method 930.15), crude protein (method 984.13), ether extract (method 920.39), crude fiber (method 962.09), crude ash (method 942.05), calcium (method 927.02), phosphorous (method 965.17), and gross energy (Parr 6400 Bomb Calorimeter, Parr Instrument, IL, USA) in the diet and fecal samples were determined using standard Association of Official Analytical Chemists methods (18). The organic matter and nitrogen-free extract (NFE) were calculated using the following equations: organic matter (%) = 100 – crude ash; NFE (%) = 100 – (crude protein + crude fiber + ether extract + crude ash). Amino acid concentrations in the diet and fecal samples were determined by high-performance liquid chromatography with post-column derivatization for the calculation of apparent total tract amino acid digestibility (19). Chromium concentrations in the feces and diets were measured using an absorption spectrophotometer (Hitachi Z-5000; Hitachi High Technologies Co., Tokyo, Japan) (20). The apparent total tract digestibility (ATTD) of energy and nutrient was calculated according to the method of a previous report (21) as illustrated in following equation: Digestibility (%) = {1 − [(Nf × Cd) / (Nd × Cf)]} × 100, where Nf and Nd represent the nutrient concentrations in feces and diet, respectively, and Cf and Cd represent the chromium concentrations in feces and diet, respectively.

### Blood sample analyses

The blood samples in the EDTA tubes were analyzed for complete blood count (CBC) using an automatic hematology analyzer (Exigo EOS hematology analyzer; Boule Medical AB, Stockholm, Sweden). The tested CBC parameters were the number of white blood cells (WBC) and their differentiation (lymphocyte, monocyte, neutrophil, and eosinophil), and the number of red blood cells (RBC) and their profiles (hemoglobin, hematocrit, mean corpuscular volume [MCV], mean corpuscular hemoglobin, mean corpuscular hemoglobin concentration [MCHC], red cell distribution width-coefficient of variation, platelet, and mean corpuscular platelet volume). The serum samples were analyzed for biochemical parameters using an automatic biochemical analyzer (Cobas C 111, Roche, Switzerland). The tested serum biochemical parameters were alanine aminotransferase (ALT), aspartate aminotransferase (AST), alkaline phosphate (ALP), total protein (TP), albumin, ammonia, creatine kinase-MB (CK-MB), lactate dehydrogenase (LDH), amylase, lipase, creatinine, blood urea nitrogen (BUN), calcium, glucose, cholesterol (CHO), and triglyceride.

### Statistical analyses

Data were analyzed using the GLM procedure of SAS (v. 9.4; SAS Institute Inc., Cary, NC, USA). The experimental unit was the Sapsaree dog. The statistical model for growth and fecal parameters, nutrient digestibility, serum biochemical parameters, and hematological profiles included life stage as the main effect. Model assumptions were evaluated using residual diagnostics. Normality of residuals and the presence of outliers were assessed using the Shapiro-Wilk test and Q–Q plots based on UNIVARIATE procedure of SAS, and homogeneity of variance was evaluated using Levene's test. No outliers were detected, and all observations were retained for the analysis. Differences among age groups were assessed using the PDIFF option. Data were presented as mean ± standard error of the mean. Frequency of hard dry feces and diarrhea were analyzed using the Chi-square test of SAS. Statistical significance was declared at *p* < 0.05, and a tendency was considered at *p* < 0.10.

## Results

### Growth and fecal parameters

The BWC was highest (*p* < 0.05) in puppies, followed by adults and seniors (Table 2). Puppies tended to have greater (*p* = 0.089) BCS at the end of the study than seniors. The DFI values (as-is and dry matter-basis) were greater (*p* < 0.05) in the puppy and adult groups than in the senior group. The DFI values standardized to MBW were highest (*p* < 0.05) in puppies, followed by adults and seniors. Consistent with this trend, a similar pattern was observed in nutrient and amino acid intake based on MBW, with puppies showing the highest values, followed by adults and then seniors. The daily fecal output (DFO) values (as-is and dry matter-basis) were greater (*p* < 0.05) in the adult group than in the other age groups. The puppy group had greater (*p* < 0.05) DFO relative to MBW than the adult and senior groups. No differences were observed on fecal score, fecal moisture, and frequency of hard dry feces and diarrhea among the age groups.

**Table 2:** Life-stage differences in growth and fecal parameters of Sapsaree dogs^1^.

Item^2^	Puppy	Adult	Senior	SEM	*p*-value
Body status					
Initial body weight, kg	19.43^c^	28.63^a^	23.83^b^	1.29	0.001
Final body weight, kg	20.22^b^	28.45^a^	23.07^b^	1.38	0.002
Body weight change, %	3.87^a^	−0.75^b^	−3.27^c^	0.61	< 0.001
Initial body condition score	5.33	6.00	5.33	0.38	0.373
Final body condition score	5.50^x^	5.00^xy^	4.17^y^	0.40	0.089
Food intake					
As-is, g/d/	459.00^a^	410.10^a^	285.76^b^	21.27	< 0.001
Dry matter, g/d/	413.81^a^	369.72^a^	257.63^b^	19.17	< 0.001
MBW, g/kg BW^0.75^/d	48.20^a^	30.64^b^	24.14^c^	1.11	< 0.001
Crude protein, g/kg BW^0.75^/d	18.71^a^	11.90^b^	9.37^c^	0.43	< 0.001
Crude fat, g/kg BW^0.75^/d	3.21^a^	2.04^b^	1.61^c^	0.07	< 0.001
Gross energy, kcal/kg BW^0.75^/d	234.73^a^	149.22^b^	117.56^c^	5.41	< 0.001
IAA, g/kg BW^0.75^/d	4.10^a^	2.60^b^	2.05^c^	0.09	< 0.001
Lysine, g/kg BW^0.75^/d	0.56^a^	0.35^b^	0.28^c^	0.01	< 0.001
Methionine, g/kg BW^0.75^/d	0.18^a^	0.12^b^	0.09^c^	0.01	< 0.001
Threonine, g/kg BW^0.75^/d	0.51^a^	0.33^b^	0.26^c^	0.01	< 0.001
DAA, g/kg BW^0.75^/d	4.57^a^	2.90^b^	2.29^c^	0.10	< 0.001
Fecal output					
As-is, g/d	57.11^b^	70.80^a^	58.39^b^	2.61	0.004
Dry matter, g/d	21.27^b^	25.64^a^	22.71^b^	0.94	0.016
MBW, g/kg BW^0.75^/d	2.51^a^	2.12^b^	2.12^b^	0.11	0.036
Fecal score	2.07	1.88	1.91	0.12	0.487
Fecal moisture, %	62.76	63.65	61.08	0.85	0.129
Frequency of hard dry feces, %	0.00	7.14	11.90		0.104
Frequency of diarrhea, %	2.38	2.38	9.52		0.246

^a^Each value is the mean of 6 replicates (one dog per cage).

^b^Puppy, under 1 year old; Adult, 1–7 years old; Senior, over 7 years old; MBW, metabolic body weight; IAA, indispensable amino acid; DAA, dispensable amino acid; Frequency of hard dry feces = (number of fecal score of less than 2 / number of cage days) × 100; Frequency of diarrhea = (number of fecal score of 3.5 or higher / number of cage days) × 100.

^a − c^Means with different superscript letters within a row are different (*p* < 0.05).

^x, y^Means with different superscript letters within a row are different (*p* < 0.10).

### Nutrient digestibility

The ATTD of dry matter was greater (*p* < 0.05) in the puppy and adult groups than in the senior group (Table 3). The ATTD of organic matter and crude protein differed (*p* < 0.05) among the age groups, being greatest in puppies, intermediate in adults, and lowest in seniors. Puppies had greater (*p* < 0.05) the ATTD of crude ash than other age dogs. The ATTD of NFE was highest (*p* < 0.05) in adults, followed by puppies and seniors. Adults and Seniors were greater (*p* < 0.05) the ATTD of calcium than puppies. Seniors had greater (*p* < 0.05) the ATTD of ether extract and crude fiber than other age dogs. The ATTD of phosphorous was highest (*p* < 0.05) in seniors, followed by adults and puppies. No difference was observed on the ATTD of gross energy among the age groups.

**Table 3:** Life-stage differences in nutrient digestibility of Sapsaree dogs^1^.

Item^2^	Puppy	Adult	Senior	SEM	*p*-value
Dry matter, %	93.78^a^	93.53^a^	92.79^b^	0.18	0.004
Organic matter, %	86.90^a^	86.34^b^	85.97^c^	0.08	< 0.001
Crude protein, %	88.86^a^	84.70^b^	83.12^c^	0.16	< 0.001
Ether extract, %	78.21^b^	78.30^b^	80.62^a^	0.36	< 0.001
Crude fiber, %	73.62^b^	74.26^b^	77.30^a^	0.28	< 0.001
Crude ash, %	55.92^a^	48.41^b^	46.76^b^	1.45	0.001
Nitrogen-free extract, %	86.74^b^	89.08^a^	81.55^c^	0.19	< 0.001
Calcium, %	77.24^b^	79.01^a^	80.42^a^	0.55	0.004
Phosphorous, %	76.79^c^	78.91^b^	79.78^a^	0.28	< 0.001
Gross energy, %	88.52	88.38	87.92	0.27	0.286

^a^Each value is the mean of 6 replicates (one dog per cage).

^b^Puppy, under 1 year old; Adult, 1–7 years old; Senior, over 7 years old.

^a − c^Means with different superscript letters within a row are different (*p* < 0.05).

In the ATTD of indispensable amino acids, the puppy and adult groups had greater (*p* < 0.05) digestibility of leucine, lysine, and threonine than in the senior group (Table 4). The ATTD of tryptophan was highest (*p* < 0.05) in puppies, followed by adults and seniors. The ATTD of histidine and methionine differed (*p* < 0.05) among the age groups, being greatest in adults, intermediate in seniors, and lowest in puppies. The ATTD of isoleucine and phenylalanine differed (*p* < 0.05) among the age groups, being greatest in adults, intermediate in puppies, and lowest in seniors. Adults had greater (*p* < 0.05) the ATTD of valine than puppies and seniors. Seniors had greater (*p* < 0.05) the ATTD of arginine than other age dogs. In the ATTD of dispensable amino acids, the puppy and adult groups had greater (*p* < 0.05) digestibility of alanine, aspartic acid, glycine, and proline than the senior group. The ATTD of glutamic acid was highest (*p* < 0.05) in puppies, followed by adults and seniors. Adults had greater (*p* < 0.05) the ATTD of cysteine than other age dogs. The ATTD of serine was highest (*p* < 0.05) in adults, followed by puppies and seniors. The ATTD of tyrosine was highest (*p* < 0.05) in adults, followed by seniors and puppies.

**Table 4:** Life-stage differences in amino acid digestibility of Sapsaree dogs^1^.

Item^2^	Puppy	Adult	Senior	SEM	*p*-value
Indispensable amino acids, %					
Arginine	96.96^b^	96.96^b^	96.79^a^	0.03	0.001
Histidine	96.88^c^	97.26^a^	97.03^b^	0.03	< 0.001
Isoleucine	96.82^b^	96.91^a^	96.72^c^	0.03	< 0.001
Leucine	96.82^a^	96.79^a^	96.62^b^	0.03	< 0.001
Lysine	96.94^a^	96.96^a^	96.79^b^	0.03	0.001
Methionine	96.86^c^	97.34^a^	97.09^b^	0.03	< 0.001
Phenylalanine	96.85^b^	96.94^a^	96.76^c^	0.02	< 0.001
Threonine	96.89^a^	96.93^a^	96.76^b^	0.03	0.001
Tryptophan	96.73^a^	97.34^b^	97.06^c^	0.03	< 0.001
Valine	96.73^b^	96.85^a^	96.66^b^	0.03	< 0.001
Dispensable amino acids, %					
Alanine	96.64^a^	96.70^a^	96.50^b^	0.03	0.001
Aspartic acid	96.82^a^	96.78^a^	96.61^b^	0.03	< 0.001
Cysteine	96.44^b^	96.77^a^	96.52^b^	0.03	< 0.001
Glutamic acid	96.85^a^	96.72^b^	96.57^c^	0.03	< 0.001
Glycine	96.80^a^	96.86^a^	96.68^b^	0.03	0.001
Proline	96.90^a^	96.97^a^	96.73^b^	0.03	< 0.001
Serine	96.82^b^	96.92^a^	96.74^c^	0.03	< 0.001
Tyrosine	97.40^c^	97.73^a^	97.54^b^	0.02	< 0.001

^a^Each value is the mean of 6 replicates (one dog per cage).

^b^Puppy, under 1 year old; Adult, 1–7 years old; Senior, over 7 years old.

^a − c^Means with different superscript letters within a row are different (*p* < 0.05).

### Serum biochemical parameters

In serum biochemistry, puppies had greater (*p* < 0.05) the levels of serum ALP, CK-MB, and glucose on day 0 than other age dogs (Table 5). The adult group had lower (*p* < 0.05) serum ammonia level on day 0 than the other age groups. In addition, adults had lower (*p* < 0.05) serum CHO level on day 0 than puppies. On day 10, the puppy group had greater (*p* < 0.05) concentrations of ALT, AST, ALP, TP, CK-MB, LDH, amylase, creatinine, BUN, calcium, CHO, and triglyceride than the other age groups. In addition, serum albumin level differed (*p* < 0.05) among the age groups, being greatest in puppies, intermediate in adults, and lowest in seniors. Serum glucose level on day 10 was highest (*p* < 0.05) in puppies, followed by seniors and adults. The adult group had lower (*p* < 0.05) serum ammonia level on day 10 than the other age groups.

**Table 5:** Life-stage differences in serum biochemical parameters of Sapsaree dogs^1^.

Item^2^	Puppy	Adult	Senior	SEM	*p*-value
Day 0					
Alanine aminotransferase, U/L	44.17	33.17	46.21	10.47	0.609
Aspartate aminotransferase, U/L	34.00	31.17	30.80	4.53	0.844
Alkaline phosphate, U/L	151.17^a^	50.67^b^	25.25^b^	36.42	0.033
Total protein, g/dL	5.82	6.48	6.03	0.39	0.349
Albumin, g/dL	4.03	4.07	3.63	0.28	0.439
Ammonia, ug/dL	149.15^a^	117.78^b^	161.95^a^	8.66	0.004
Creatine kinase-MB, U/L	256.68^a^	87.65^b^	101.86^b^	16.20	< 0.001
Lactate dehydrogenase, U/L	51.50	40.50	59.40	7.85	0.234
Amylase, U/L	680.87	776.58	717.28	86.62	0.639
Lipase, U/L	33.33	49.00	39.20	9.65	0.467
Creatinine, mg/dL	0.33	0.44	0.39	0.05	0.168
Blood urea nitrogen, mg/dL	25.65	24.27	19.71	2.34	0.188
Calcium, mg/dL	11.64	10.12	12.98	1.84	0.531
Glucose, mg/dL	106.33^a^	94.67^b^	94.50^b^	2.83	0.005
Cholesterol, mg/dL	244.50^a^	170.67^b^	212.00^ab^	18.60	0.015
Triglyceride, mg/dL	45.17	46.00	56.25	5.98	0.332
Day 10					
Alanine aminotransferase, U/L	59.33^a^	16.33^b^	21.50^b^	7.38	0.002
Aspartate aminotransferase, U/L	35.17^a^	18.17^b^	16.17^b^	2.02	< 0.001
Alkaline phosphate, U/L	149.33^a^	24.00^b^	21.50^b^	28.19	0.008
Total protein, g/dL	6.02^a^	3.57^b^	2.90^b^	0.24	< 0.001
Albumin, g/dL	4.38^a^	2.45^b^	1.88^c^	0.18	< 0.001
Ammonia, ug/dL	186.40^a^	129.20^b^	166.58^a^	9.50	0.002
Creatine kinase-MB, U/L	231.89^a^	52.04^b^	57.09^b^	8.50	< 0.001
Lactate dehydrogenase, U/L	73.17^a^	29.83^b^	27.17^b^	3.63	< 0.001
Amylase, U/L	656.45^a^	400.53^b^	349.08^b^	39.39	< 0.001
Lipase, U/L	36.50	29.83	29.00	5.15	0.540
Creatinine, mg/dL	0.91^a^	0.56^b^	0.63^b^	0.05	< 0.001
Blood urea nitrogen, mg/dL	60.25^a^	34.23^b^	36.53^b^	4.09	0.001
Calcium, mg/dL	11.34^a^	5.86^b^	5.87^b^	0.28	< 0.001
Glucose, mg/dL	100.17^a^	57.00^c^	73.33^b^	3.73	< 0.001
Cholesterol, mg/dL	148.50^a^	54.33^b^	73.83^b^	9.27	< 0.001
Triglyceride, mg/dL	43.83^a^	23.00^b^	26.83^b^	3.10	0.001

^1^Each value is the mean of 6 replicates (one dog per cage).

^2^Puppy, under 1 year old; Adult, 1–7 years old; Senior, over 7 years old.

^a − c^Means with different superscript letters within a row are different (*p* < 0.05).

### Hematological profiles

On day 0, the number of white blood cells and differential percentages did not differ among age groups (Table 6). In erythrocyte-related profiles, the number of red blood cells and levels of hemoglobin and hematocrit were greater (*p* < 0.05) in adults than other age dogs. Puppies tended to have greater (*p* = 0.083) MCV level than adults. The Adult group had lower (*p* < 0.05) platelet level than the other age groups. On day 10, puppies had greater (*p* < 0.05) the number of white blood cells than other age dogs. Puppies had greater (*p* < 0.05) lymphocyte percentages than seniors, but tended to lower (*p* = 0.090) neutrophil percentages. In erythrocyte-related profiles, the number of red blood cells and hemoglobin level were greater (*p* < 0.05) in the adult group than in the other age groups. Puppies tended to have greater (*p* = 0.052) MCV level than adults. Puppies had lower (*p* < 0.05) MCHC level than other age dogs.

**Table 6:** Life-stage differences in hematological profiles of Sapsaree dogs^1^.

Item^2^	Puppy	Adult	Senior	SEM	*p*-value
Day 0					
White blood cell, × 10^9^/L	11.03	10.38	10.02	1.35	0.867
Lymphocyte, %	29.82	24.75	23.50	2.13	0.119
Monocyte, %	8.73	7.38	8.45	0.67	0.350
Neutrophil, %	56.57	62.47	62.65	2.48	0.178
Eosinophil, %	4.08	4.66	4.72	1.19	0.917
Basophil, %	0.80	0.74	0.68	0.54	0.989
Red blood cell, × 10^9^/L	7.00^b^	8.37^a^	7.21^b^	0.25	0.003
Hemoglobin, g/dL	15.13^b^	17.90^a^	15.57^b^	0.52	0.004
Hematocrit, %	40.72^b^	46.73^a^	40.62^b^	1.60	0.025
MCV, fL	58.23^x^	54.25^y^	56.10^xy^	1.16	0.083
MCH, pg	21.66	20.80	21.53	0.40	0.295
MCHC, g/dL	37.21	38.33	38.40	0.48	0.182
RDW-CV, %	14.87	15.05	14.92	0.20	0.730
Platelet, × 10^9^/L	338.83^a^	215.00^b^	304.67^a^	32.95	0.047
MPV, fL	5.98	6.05	6.02	0.24	0.981
Day 10					
White blood cell, × 10^9^/L	11.97^a^	9.52^b^	8.10^b^	0.60	0.001
Lymphocyte, %	31.98^a^	27.37^ab^	24.60^b^	1.79	0.032
Monocyte, %	8.73	9.00	7.68	0.67	0.363
Neutrophil, %	53.57^y^	57.27^xy^	61.95^x^	2.49	0.090
Eosinophil, %	4.94	5.55	5.02	0.84	0.856
Basophil, %	0.78	0.81	0.75	0.65	0.997
Red blood cell, × 10^9^/L	6.62^b^	8.43^a^	6.98^b^	0.30	0.002
Hemoglobin, g/dL	13.97^b^	17.37^a^	14.95^b^	0.58	0.003
Hematocrit, %	39.45	40.55	40.13	4.47	0.985
MCV, fL	59.85^x^	55.68^y^	57.55^xy^	1.09	0.052
MCH, pg	21.13	20.58	21.47	0.37	0.266
MCHC, g/dL	35.35^b^	37.02^a^	37.37^a^	0.55	0.045
RDW-CV, %	15.50	15.33	15.23	0.26	0.773
Platelet, × 10^9^/L	290.50	236.33	294.67	45.24	0.606
MPV, fL	6.63	6.47	6.40	0.35	0.888

^1^Each value is the mean of 6 replicates (one dog per cage).

^2^Puppy, under 1 year old; Adult, 1–7 years old; Senior, over 7 years old; MCV, mean corpuscular volume; MCH, mean corpuscular hemoglobin; MCHC, mean corpuscular hemoglobin concentration; RDW-CV, red cell distribution width-coefficient of variation; MPV, mean platelet volume.

^a − c^Means with different superscript letters within a row are different (*p* < 0.05).

^x, y^Means with different superscript letters within a row are different (*p* < 0.10).

## Discussion

The present study identified age-related differences in growth and fecal parameters, nutrient digestibility, serum biochemical parameters, and hematological profiles in Sapsaree dogs fed the same food. By maintaining the same food composition across age groups, the observed differences were found to be primarily due to physiological and metabolic changes associated with growth, maintenance, and aging.

Maintaining an appropriate BW and BCS is important for long-term health and longevity of companion dogs. Because BW and BCS are the most practical indicators available to owners and clinicians, changes in these parameters provide important insight into the adequacy of dietary energy supply and overall physiological condition. In this study, age-related differences in BWC and BCS were observed despite the application of a standardized feeding protocol based on the MER. Puppies exhibited increase in BWC, whereas adult and senior dogs showed reductions in BWC over the experimental period. These contrasting responses are consistent with life-stage physiological priorities, with puppies focusing their nutrients on growth and tissue formation, whereas adult and senior dogs primarily focus on maintaining existing body tissues (2, 4, 17). However, individual variations in MER cannot be excluded. Additionally, providing dietary energy levels recommended by the NRC was shown to maintain BW gain in Sapsaree puppies without causing excessive increases in BCS during the study. Meanwhile, the reduction in BWC observed in senior dogs may indicate a transient shift in energy utilization. This change was consistent across individual seniors, suggesting a group-level response. Overall, these results appear to reflect normal biological variation in energy utilization and/or short-term adaptation to experimental conditions. Moreover, the relatively lower BCS observed in senior dogs appears to reflect age-related changes in nutrient utilization efficiency. However, all BCS values remained within or close to the optimal range (22), suggesting that the experimental food system maintained adequate body condition across all age groups.

Since the provided food amounts were calculated based on the MER, differences in DFI values may be attributable to the MER-based strategy rather than voluntary decrease in food intake. Nevertheless, although the DFI of puppies and adults was similar, their BWC patterns were different, indicating age-related differences in physiological priorities and metabolic requirements. This discrepancy between DFI and BWC indicates that differences in energy consumption and distribution according to life stage play a crucial role, suggesting that even with the MER-based diet, net energy balance can vary depending on physiological state. In particular, variation in basal metabolic rate and overall energy expenditure may contribute to differences in BWC responses by altering the allocation of ingested energy among competing physiological processes (23, 24). Meanwhile, quantitative differences in DFO values were observed when expressed on an as-is and dry matter basis. However, when DFO was normalized to MBW, the pattern of differences among age groups changed, indicating that part of changes in fecal output was attributable to differences in body size. These contrasting results suggest that differences in absolute fecal output were largely influenced by body size, whereas DFO relative to MBW reflects metabolic and physiological differences associated with life stage. Fecal parameters such as fecal score, moisture content, and frequency of constipation or diarrhea did not differ across age groups. These findings indicate that fecal quality remained stable across age groups. Taken together, our results demonstrate that dogs at different life stages respond differently to standardized MER-based feeding system, reflecting age-related differences in the priority of growth and maintenance as well as metabolic regulation.

The ATTD of nutrients and amino acids varied across age groups, indicating that life-stage influences nutrient utilization even when dogs are fed the same diet. Because digestibility estimates depend on energy intake relative to physiological requirements, feeding based on MER may have introduced differences in DFI across life stages, which could influence nutrient digestibility. In our study, puppies and adult dogs showed higher dry matter digestibility than senior dogs, while the digestibility of crude protein reduced with age. This is also consistent with previous studies on age-related changes in protein metabolism and digestive physiology (1, 25). In growing dogs, protein accumulation for body tissue formation occurs actively, reflecting a physiological state associated with growth (26, 27). However, ATTD values reflect not only undigested dietary protein but also endogenous nitrogen losses, including digestive secretions and epithelial cell turnover (28). Therefore, the higher crude protein digestibility observed in puppies reflects age-related differences in digestive capacity and the relative contribution of endogenous nitrogen excretion. Amino acid digestibility also showed distinct differences by age, with adult dogs having the highest or similar levels to puppies for the ATTD of most essential and non-essential amino acids, while senior dogs showing relatively lower values. These results suggest that the relatively high amino acid digestibility in adult dogs is since digestive enzyme secretion and intestinal absorption capacity are known to remain relatively stable during the maturation stage after growth (2). On the other hand, the lower amino acid digestibility observed in senior dogs may be related to age-related changes in digestive physiology, such as changes in intestinal structure and decreased cell proliferation (29). However, because amino acid digestibility in this study was assessed using ATTD methods, these results should be considered with the limitation of this approach. Since post-ileal microbial activity and endogenous nitrogen losses can affect fecal amino acid recovery (28), the results of this study represent overall apparent availability. Collectively, these findings suggest that protein utilization may vary across ages and that the ATTD of crude protein does not necessarily reflect the digestibility patterns of individual amino acids.

The ATTD of ether extract, crude fiber, and NFE showed distinct age-related patterns in this study. Ether extract and crude fiber digestibility were higher in senior dogs than in puppies and adults, but NFE digestibility was greatest in adults and lowest in seniors. These findings appear to reflect differences in the digestive mechanisms of these nutrient fractions. NFE represents readily digestible carbohydrates, such as starch, that are enzymatically digested and absorbed in the small intestine, whereas crude fiber is more resistant to enzymatic digestion and is mainly fermented by gut microbiota in the large intestine (30, 31). Thus, the lower NFE digestibility observed in senior dogs may indicate reduced efficiency of small intestinal carbohydrate digestion with advancing age. In contrast, the higher crude fiber digestibility in senior dogs may reflect differences in fiber utilization and hindgut digestive processes, potentially influenced by life-stage-related changes in gut microbiota composition. Age-related differences in gut microbiota composition have also been reported in Sapsaree dogs (32), which may contribute to these observations. Additionally, ether extract digestibility followed a similar pattern to crude fiber, being greater in senior dogs than in puppies and adults. It may be related to differences in lipid digestion efficiency, bile acid metabolism, or digesta transit characteristics depending on life stage. Furthermore, interactions between dietary fiber and lipid digestion, such as fiber-mediated alterations in bile acid metabolism and lipid absorption, may also contribute to these patterns (33, 34), but further investigation is required to clarify these mechanisms.

The ATTD of mineral components, including crude ash, calcium, and phosphorous, also differed among age groups. The observed results are interpreted as age-related differences in the efficiency of mineral utilization and excretion rather than changes in intestinal absorptive capacity. These differences may reflect life-stage variations in mineral utilization and retention associated with growth and skeletal development (growth vs. maintenance stages), as growing animals exhibit increased mineral deposition in bone during skeletal growth (35, 36). Taken together, these findings demonstrate that aging alters the relative efficiency of digestive processes across nutrients, which should be considered when evaluating subsequent age-dependent physiological responses.

Serum biochemical parameters showed age-related differences in the present study. Similar age-related differences in serum biochemical parameters have also been reported in Sapsaree dogs, indicating that metabolic profiles vary across ages (37). Importantly, the observed differences were consistent in both direction and magnitude with previously reported age-related physiological changes in dogs (17), suggesting that they reflect normal metabolic adaptations rather than pathological alterations. These findings indicate that the changes are biologically meaningful within a physiological context, reflecting age-related metabolic regulation. In particular, puppies showed greater activities of hepatic enzymes and higher concentrations of circulating protein- and lipid metabolites than adult and senior dogs. These results may be interpreted as reflecting the greater metabolic activity to support growth and tissue development during early life. In this regard, serum ALP levels in puppies were greater than that in adults and seniors on day 0 and 10. It appears to reflect the increased bone turnover associated with rapid growth, as elevated ALP levels in puppies has been associated to enhanced bone formation and remodeling during skeletal development (3). Additionally, the higher phosphorous metabolism required to maintain positive phosphorous balance during growth may further contribute to the increased ALP levels in puppies (38). Furthermore, serum albumin concentrations decreased with age, with the highest values observed in puppies and the lowest in senior dogs. This may be attributed to life-stage differences in protein metabolism and amino acid utilization, which are consistent with previous reports of age-related variation in serum biochemical parameters and nutrient digestibility in dogs (10, 39, 40). Meanwhile, pancreatic enzyme levels (amylase and lipase) in serum were also high in puppies. Although circulating digestive enzyme activity does not directly reflect enzymatic secretion, these results may be related to the developmental maturation of digestive enzyme activity during early life (41), which could contribute to the higher nutrient digestibility observed in younger dogs.

Ammonia concentrations were higher in puppies and senior dogs than in adults. Since ammonia is a nitrogenous product generated during amino acid deamination (42, 43), these findings suggest that there were differences in nitrogen turnover and amino acid metabolism depending on age. These patterns are consistent with the differences in ATTD of crude protein, suggesting that age-related variation in protein digestion and utilization may influence systemic nitrogen metabolism. In addition, the reduction in BWC in senior dogs suggests a potential negative energy balance, which may promote protein catabolism under conditions of insufficient energy intake (44). Because ammonia is generated during amino acid catabolism, this process may influence ammonia production (26). Thus, ammonia concentration may reflect not only age-related metabolic differences but also energy balance status of the animals. Taken together, our results suggest that age-related changes in metabolic activity and digestive function, together with differences in energy balance, may contribute to differences in serum biochemical profiles and nutrient utilization among life stages in Sapsaree dogs.

In hematological parameters, at baseline and at the end of the experiment, leukocyte-related parameters (WBC and lymphocyte counts) were generally higher in puppies than in adult and senior dogs. This may reflect age-related variation in immune cell distribution, as leukocytes are involved in the immune responses (45). Previous studies have also reported that hematological parameters vary with age in dogs (10, 37). In contrast, erythrocyte-related indices such as RBC and hemoglobin concentrations were higher in adults than in puppies and seniors, suggesting relatively stable erythropoietic status after maturity (46, 47). MCV values were slightly outside the reported reference range (60.0–72.0 fL) across all age groups, whereas other hematological parameters remained within normal physiological limits. Because hematological reference range can vary depending on factors such as age, sex, breed, size, or analytical methodology (8, 10, 48), the observed MCV values appear to reflect biological variation rather than clinically relevant hematological abnormalities. Overall, these findings indicate that although several hematological parameters differed among ages, the values were generally within physiological ranges and did not have any adverse health consequences.

This study has several limitations that should be considered. First, food amounts were determined based on MER, which may have influenced DFI across life stages and could affect nutrient digestibility and DFO. Second, dietary fiber was evaluated using crude fiber, and total dietary fiber, including soluble and insoluble fractions, was not analyzed, limiting the assessment of fiber-related physiological responses. In addition, since amino acid digestibility was evaluated using the ATTD methods, the results reflect the limitations of this approach. Third, the muscle condition score, an important indicator for assessing muscle mass and overall nutritional status (49), was not assessed in this study, which would enable a more comprehensive evaluation. Finally, the use of a single breed and broad age classification may limit generalizability. Future studies incorporating more diverse populations, refined age stratification, and integrative analyses such as gut microbiota and metabolites profiling are warranted to provide deeper insight into life-stage-specific physiological responses.

In conclusion, this study demonstrated that nutrient digestibility and physiological responses differ across life stages in Sapsaree dogs. Puppies generally showed greater the ATTD of crude protein and several nutrients, whereas adult dogs exhibited relatively higher the ATTD of many amino acids, suggesting age-dependent differences in nutrient digestive patterns. Life-stage differences were also determined in nitrogen metabolism, as reflected by changes in crude protein digestibility and serum ammonia concentrations, indicating that both digestive processes and energy balance contribute to systemic metabolic responses. Serum biochemical and hematological parameters also varied among ages, reflecting differences in metabolic activity associated with growth and maturation. However, most values remained within normal physiological ranges, indicating that these variations appear to represent normal life-stage-related physiological differences. The important point is that although the same food was fed to all dogs, the feeding amounts were determined based on MER, which may have contributed to differences in metabolic and physiological responses among age groups. Collectively, our findings suggest that standardized feeding approach based on MER may not fully account for age-related differences in digestive and metabolic responses. In addition, further research is needed to develop more precise and age-specific food formulations to improve the long-term health and wellbeing of Sapsaree dogs.

## Data Availability

The original contributions presented in the study are included in the article/supplementary material, further inquiries can be directed to the corresponding authors.
